# Age and Living Situation as Key Factors in Understanding Changes in Alcohol Use during COVID-19 Confinement

**DOI:** 10.3390/ijerph182111471

**Published:** 2021-10-31

**Authors:** Víctor J. Villanueva-Blasco, Verónica Villanueva Silvestre, Andrea Vázquez-Martínez, Antonio Rial Boubeta, Manuel Isorna

**Affiliations:** 1 Faculty of Health Sciences, Valencian International University, 46002 Valencia, Spain; avazquezm@universidadviu.com; 2 Faculty of Psychology, University of Santiago de Compostela, 15782 Santiago de Compostela, Spain; antonio.rial.boubeta@usc.es; 3 Faculty of Education and Social Work, Campus as Lagoas, University of Vigo, 32004 Ourense, Spain; isorna.catoira@uvigo.es

**Keywords:** alcohol, risky consumption, COVID-19, confinement measures, age, living situation

## Abstract

(1) The aim of the present study was to evaluate and characterize changes in alcohol use during the COVID-19 confinement in a sample of Spanish adults, analyzing their age and living situation as defining life cycle variables. (2) Method: Data from 3779 individuals were collected through a set of online surveys. AUDIT-C was used to measure the frequency of consumption, the average daily consumption, intensive consumption, risky consumption, and Standard Drink Units. (3) Results: Although alcohol consumption during confinement showed a significant general decline, age revealed important differences, with the decline being more pronounced in adults from 18 to 29 years old. The living situation also showed significant differences. The largest decreases in alcohol consumption were found in those who lived with their parents or other relatives, whereas those who lived alone or with a partner even increased their level of consumption. In addition, the data show a significant interaction between these two variables and gender. (4) Conclusions: Age and cohabitation processes are key factors in understanding the life situation of each individual during confinement and, consequently, in explaining consumption patterns. The results obtained provide interesting recommendations for designing prevention policies in both normal and crisis circumstances, emphasizing the need to understand alcohol use from a psychosocial perspective.

## 1. Introduction

In March 2020, unprecedented measures were adopted to control the COVID-19 pandemic, including social distancing and mobility restrictions, which meant confining a large part of the population to their homes. In this context, there was an increase in stimuli that produce psychosocial stress [1], as well as modifications in habits and routines in different areas of life, including drug use [2,3,4]. The abuse of legal and illegal drugs became a secondary issue compared to the health risks directly related to COVID-19, even though its impact on health has been well established. Alcohol is the most widely consumed substance in the world, the main public health problem, and the cause of serious social and economic harm [5]. We know that some people use it as a coping mechanism in response to stressful events and social crises [6], but research has also established that it weakens the immune system [7] and increases the risk of viral infections [8].

In this regard, literature that has analyzed the changes during the confinement period and immediately afterwards has found changes in both the prevalence and pattern of alcohol consumption [9,10,11,12]. Some studies indicate that, during confinement, the prevalence of alcohol consumption, the frequency of consumption, or the number of drinks on each occasion stayed the same or declined compared to pre-pandemic levels [11,13,14]. Other studies note that the decrease in prevalence and risky drinking was more significant in people from 18 to 29 years old [10,11,15,16,17,18]. However, in this same age group, an increase in alcohol consumption, in terms of frequency and average amount, has also been reported [19,20], indicating that this could be a strategy used to cope with the stressful emotional burden of the pandemic [19]. Moreover, other studies report that, whereas part of the drinking population showed no change or less intake, another part showed an increase in consumption [21,22,23]. Finally, other studies report increases in alcohol consumption [24,25] and risky drinking [16], mostly in adults from 30 to 64 years old [14,26], with gender differences [22,27].

Thus, age seems to play a relevant role in the impact of isolation and mobility restriction measures on the social dynamics associated with alcohol consumption. In Spain, the predominant pattern of alcohol consumption is associated with spending leisure time in social groups (friends, colleagues, relatives, etc.). Therefore, the closing of bars, restaurants, clubs, and pubs, the usual places for alcohol consumption, led to changes in the availability of alcohol, which could contribute to reducing its use and corresponding harm [6]. However, the fact that alcohol consumption was confined exclusively to the home [22] may have created a situation of greater vulnerability to drinking alcohol in people with a higher level of dependence [10,28], mainly older adults and females, because at-home drinking is a strong predictor of risky consumption [29,30,31].

Likewise, alcohol consumption during the confinement period may also have been mediated by the family situation and structure. Some mothers and fathers were teleworking and caring for their children at the same time, and others were temporarily or permanently unemployed, all of which contributed to greater stress due to the duration of the situation and the extent of the consequences [32]. Thus, in both cases, the increase in stress may have led to increased alcohol consumption as a coping strategy [33]. In addition, alcohol consumption could also be used to reduce unpleasant feelings specifically related to isolation or loneliness [34], especially in people who live alone during confinement [35].

The aim of the present cross-sectional study was not only to evaluate and characterize possible changes in alcohol consumption due to confinement, as other studies have suggested, but also to analyze to what extent Age and Living Situation could be key variables in explaining these changes. Despite the inherent limitations of the methodological design, the relatively large sample size and broad age range should help to better understand the consumption changes experienced, after examining the lifecycle stage and situation in which each individual is immersed. The results derived from the present study should be interpreted from a psychosocial perspective, with important implications for prevention.

## 2. Materials and Methods

### 2.1. Design

This study is descriptive and non-probabilistic, and it uses convenience sampling. A battery of online surveys was used to collect and evaluate the variables under study. Age ranges were established based on those found to have adequate internet access, as stated in the Equipment and Use of Information and Communication Technologies at Home Survey [35].

### 2.2. Population

The initial sample included 4213 participants. Of them, 434 (10.3%) were removed because of missing values, incoherent response patterns, or not being within the established age range (18–64 years old). The final sample contains data from 3779 participants (70% female; 30% male) with an average age of 37.76 years (SD = 11.95), corresponding to 17 autonomous regions and the two Spanish autonomous cities.

By age range, 14.7% (*n* = 558) are from 18 to 24 years old (56.6% female; 43.4% male); 17.3% (*n* = 656) from 25 to 29 years old (62.7% female; 37.3% male); 13.8% (*n* = 522) from 30 to 34 years old (49% female; 51% male); 23.8% (*n* = 900) from 35 to 44 years old (45.6% female; 54.4% male); 19% (*n* = 717) from 45 to 54 years old (47.3% female; 52.7% male); and 11.3% (*n* = 427) from 55 to 64 years old (37% female; 63% male).

Regarding employment, 47.4% (*n* = 1142) have a full-time job, 8.4% (*n* = 203) have a part-time job, 7.7% (*n* = 186) are self-employed, 9.7% (*n* = 235) have a job covered by a Temporary Employment Regulation Plan (ERTE), 1% (*n* = 23) are homemakers, 14.7% (*n* = 355) are students, 1.3% (*n* = 31) are pensioners or retirees, 8.9% (*n* = 215) are unemployed, and 0.9% (*n* = 21) chose to leave this question blank.

The database was weighted to correct for the bias introduced due to the intentional non-probabilistic nature of the sampling, which translated into a sampling imbalance in the participants’ gender.

### 2.3. Procedure

Data collection started on 14 April 2020, after the first 30 days of confinement measures, and it ended on 29 May when the de-escalation measures started. The data collection strategy was based on a survey hosted on a web, posts on social media, and advertisements via e-mail and smartphone messaging applications. Participants were informed that participation was voluntary, in accordance with the Spanish Organic Law 3/2018 on Personal Data Protection and Digital Rights Guarantee [36]. They were asked to give their consent to participate. Selection criteria were: (a) age between 18 and 64 years old; (b) explicit agreement to participate; and (c) properly filling out the survey.

### 2.4. Study Variables

The sociodemographic variables considered were: (a) gender (male, female); (b) age, according to the age ranges established in the EDADES survey [37] (18–24 years, 25–29 years, 30–34 years, 35–44 years, 45–54 years, 55–64 years); and (c) living situation: (1) lives alone; (2) lives with parents or other relatives; (3) lives with a partner; (4) shares a flat with people who are neither relatives nor a partner; (5) another living situation.

The AUDIT-C [38], a short version of the Alcohol Use Disorders Identification Test, was used to measure alcohol consumption. The AUDIT-C is composed of three items that analyze consumption frequency, average daily consumption, and frequency of intensive consumption. Frequency of consumption (average number of drinking days per month) was measured with the question “How often do you consume alcoholic drinks?”, with possible answers being: (0) Never; (1) Once a month or less; (2) 2 to 4 times a month; (3) 2 to 3 times a week; (4) 4 or more times a week. Daily average consumption was measured with the question “How many alcoholic drinks do you usually have on a normal day?”, with possible answers being: (0) 1 or 2; (1) 3 or 4; (2) 5 or 6; (3) 7, 8, or 9; and (4) 10 or more. Intensive consumption, characterized by high alcohol intake in a short period of time, was measured with the question “How often do you drink 6 or more alcoholic beverages in a single day?”, with possible answers being: (0) Never; (1) Less than once a month; (2) Monthly; (3) Weekly; (4) Daily or almost daily.

Risky consumption is defined as a pattern of consumption that increases the risk or likelihood of harmful consequences for the consumer, even when the consumer does not have any current disorders [39]. The limit of risky consumption was established at 4 points or more in females and 5 or more in males, based on the total score on the AUDIT-C [40,41].

Furthermore, the Spanish Standard Drink Unit (SDU), equivalent to 10 g of pure alcohol, according to which 1 fermented beverage (beer, wine) = 1 SDU and 1 distilled beverage (spirit, liquor) = 2 SDUs (40), was used. Because this is a standard measure, the amount of alcohol ingested in a day can be recorded more accurately. A Likert-type response scale with six options was used: (0) 1 or 2; (1) 3 or 4; (2) 5 or 6; (3) 7, 8, or 9; and (4) 10 or more. Participants were given exact information about the SDU equivalencies.

The difference between the pre-COVID and confinement scores was also calculated for the AUDIT-C, as well as for each of its individual items and the SDU. A negative score indicates an increase in consumption, a positive score indicates a decrease, and zero indicates no change.

Participants were asked about these drinking variables in relation to the confinement period (April–May 2020) and retrospectively in relation to their drinking during the six months prior to the pandemic (March 2020).

### 2.5. Statistical Analysis

Data analysis was performed with the IBM SPSS Statistics for Windows, version 25. As a first step, the sample was weighted as a balancing strategy. After that, intragroup differences were examined through a frequency analysis and chi square test (disaggregated according to age) of the frequency of consumption, average daily consumption, intensive consumption, and SDUs per day before and during confinement. To compare measures of these variables, as well as the score on the AUDIT-C, to establish alcohol consumption before and during the pandemic, compliance with the normality criterion (Kolmogorov−Smirnov) and homoscedasticity (Levene’s equal variances) was checked, considering gender as the independent variable by applying a Student’s T-test.

Comparisons of between-group means before and during confinement were also carried out. For independent samples, a Student’s T-test was performed to analyze differences between the different age groups. To obtain a measure of the effect size, chi square and Cohen’s d were used.

Comparison of means was also performed to test for significant differences between groups before and during confinement. Specifically, analysis of variance (ANOVA) was used to test for differences between age groups, using Bonferroni for post hoc tests and eta squared to calculate the effect size.

Finally, several analyses of variance (ANOVA) were performed to study the interaction effect between the three AUDIT-C variables, the SDU measurement before and during the confinement, and the age variable, subsequently including gender and living situation.

### 2.6. Ethical Aspects

The study was carried out in accordance with The Code of Ethics of the World Medical Association (Declaration of Helsinki) and approved by the Committee of Evaluation and Follow-up of Research with Human Beings (CEISH) from Valencian International University (VIU).

## 3. Results

Of the total sample (*n* = 3779), 62% of the participants (*n* = 2345) had consumed alcohol in the past six months; 46.65% of them (*n* = 1094) were female, and 53.35% (*n* = 1251) were male; 17.6% (*n* = 412) were from 18 to 24 years old; 21.1% (*n* = 493) from 25 to 29 years old; 14.3% (*n* = 334) from 30 to 34 years old; 22.3% (*n* = 523) from 35 to 44 years old; 16.7% (*n* = 392) from 45 to 54 years old; and 8% (*n* = 188) from 55 to 64 years old.

Table 1 presents the changes in the pattern of alcohol consumption, comparing the measures before the pandemic and during confinement and showing how much consumption decreased, stayed the same, or increased for the different study variables. Because 2.6% (*n* = 63) showed missing values that did not allow these differences to be established, they were not considered in the analyses.

For the total sample of alcohol consumers (Table 1), in relation to the mean AUDIT-C score, 49.5% showed a decrease, 33.8% maintained the same score, and 16.7% increased their score. The greatest decrease is observed in the frequency of consumption: 36.2% showed a decrease, 45.4% maintained the same score, and 18.3 % increased their score. In relation to the frequency of intensive consumption, 29.3% showed a decrease, 66.4% maintained the same score, and 4.3% increased their score. Average daily consumption showed less variation between before and during confinement, where 14.2% showed a decrease, 82.8% maintained the same score, and 3% increased their score, as did the mean number of SDUs per day, where 7.5% showed a decrease, 88.6% maintained the same score, and 3.9% increased their score.

When analyzing the data according to age (Table 1), a common pattern is observed for all the alcohol consumption variables studied, with greater decreases in the younger age ranges. Although these decreases continue in older ages, they are smaller as the age increases. The inverse trend is observed for increases in consumption, with results showing that, as age increases, the percentage of consumers who increase their consumption is higher, except in the case of the frequency of intensive consumption, which shows a more heterogeneous pattern.

To further characterize the changes observed in Table 1, additional analyses were conducted to determine the percentage of alcohol consumers before and during confinement for each of the responses to the four drinking variables analyzed.

Thus, the frequency of alcohol consumption was multiplied by a factor of 11 in those who did not consume alcohol in the 18 to 24 age range and decreased with age (multiplied by a factor of 2.3 in the 55 to 64 age range). However, the results also showed that, during confinement, the percentage of people who consume alcohol four or more days a week increased. This subsample represents 11.64% (*n* = 273) of the total number of alcohol consumers before the pandemic, increasing to 17.57% (*n* = 412) during confinement. That is, the percentage of people who consume alcohol four or more days a week increased by a factor of 1.5 during confinement, corresponding to 5.2% of 18- to 24-year-olds (1.67 times higher); 7.3% of 25- to 29-year-olds (1.9 times higher); 8.3% of 30- to 34-year-olds (1.9 times higher); 22.9% of 35- to 44-year-olds (1.77 times higher); 30.8% of 45- to 54-year-olds (1.36 times higher); and 25.6% of 55- to 64-year-olds (1.17 times higher).

With regard to average daily alcohol consumption, before confinement, 80% consumed between one and two alcoholic beverages per day; 15% consumed three to four; 3.7% consumed five to six; and 1.3% consumed seven to nine alcoholic beverages per day. During confinement, a decrease in average daily alcohol consumption was observed in all the age ranges, with the majority (90.5%) consuming between one and two alcoholic beverages per day. This tendency was more pronounced in consumers in the lower age ranges (18–29 years) than in those in the higher age ranges.

Regarding the prevalence of intensive alcohol consumption, before confinement, 58.1% never showed this pattern; 26.6% did so less than once a month; 9.8% monthly; 5% weekly; and 0.5% daily or almost daily. During confinement, intensive drinking decreased in all the age ranges, which meant that 83.6% now reported not having a heavy drinking pattern. In other words, intensive alcohol consumption decreased 1.44 times compared to before the pandemic.

With regard to SDUs, results show that, during confinement, the lower age ranges (18–24 years, 25–29 years, and 30–34 years) showed a decrease in all the SDU indicators per day, except 1 or 2 SDUs per day, which increased. However, for the higher age ranges (35–44 years, 45–54 years, and 54–64 years), in general terms, the prevalence rates remained at pre-confinement levels.

**Figure 1:**
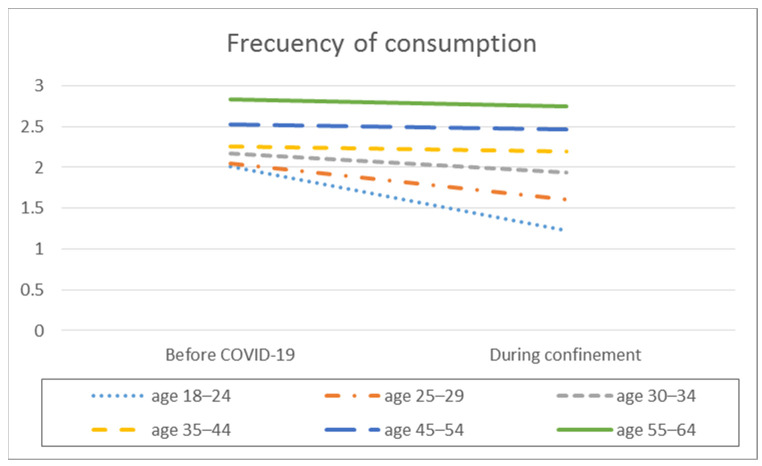
Average frequency of consumption by age before the pandemic and during COVID-19 confinement.

**Figure 2:**
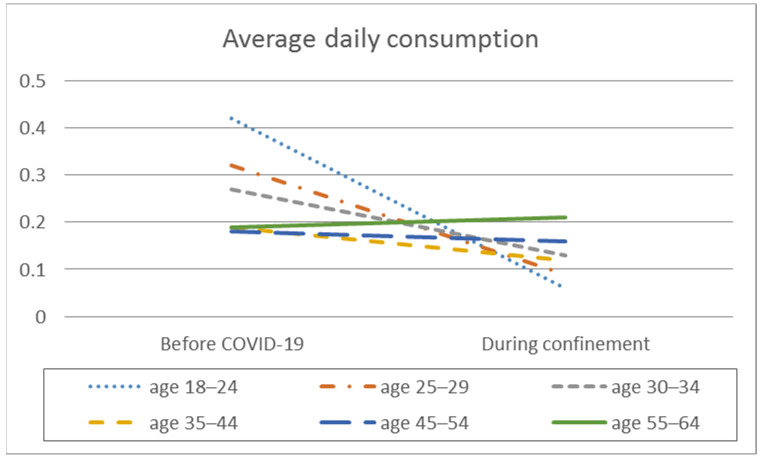
Average daily alcohol consumption by age before the pandemic and during COVID-19 confinement.

**Figure 3:**
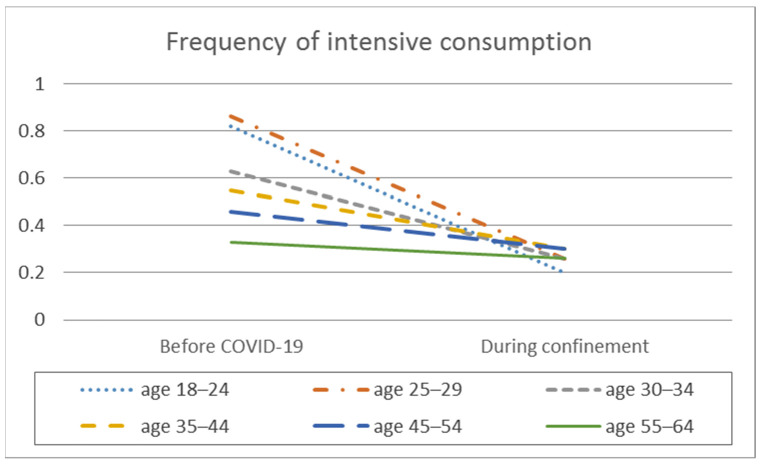
Frequency of intensive alcohol consumption by age before the pandemic and during COVID-19 confinement.

**Figure 4:**
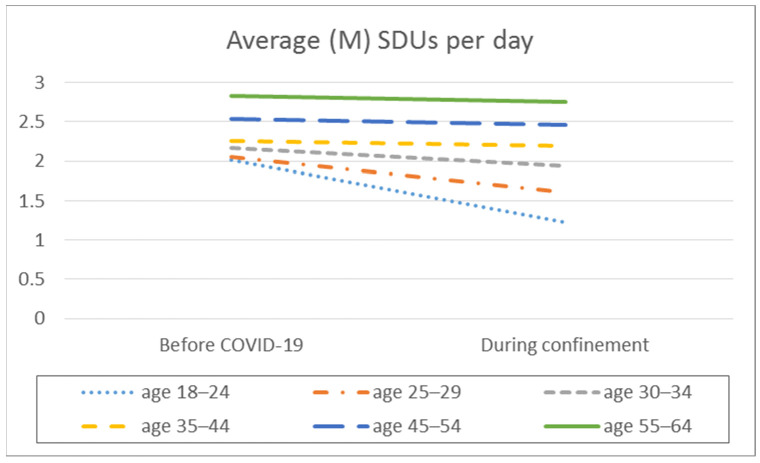
Average number of SDUs per day by age before the pandemic and during COVID-19 confinement.

In the analysis of the difference in means, significant intra-group differences are observed in the frequency of consumption, the average daily alcohol consumption, the frequency of intensive alcohol consumption, and the average number of SDUs consumed per day, but differentiated depending on the age range (Table 2, Figure 1, Figure 2, Figure 3 and Figure 4).

The lower age ranges (18–24 years, 25–29 years, and 30–34 years) showed higher consumption frequencies before the pandemic than during confinement (*p* < 0.001), with a medium effect size in the younger age ranges and a small effect size in the other groups. These same age groups, as well as the 35 to 44 age group, showed higher average daily consumption differences before confinement than during confinement (*p* < 0.001), with a medium effect size for the 18 to 24 age group and a small effect size for the others. In contrast, the 45 to 64 age group showed similar frequencies of monthly and average daily alcohol consumption before and during confinement.

In all the age groups, with the exception of the oldest group from 54 to 65 years old, there was a decrease in the frequency of intensive alcohol consumption (six or more alcoholic drinks in a single day) during confinement compared to before confinement (*p* < 0.001), with medium effect sizes in the 18 to 44 age range and small effect sizes in the 45 to 54 age range. That is, as the age decreases, the effect size is larger.

Regarding the mean number of SDUs consumed per day, the 18- to 24-year-old and 25- to 29-year-old age ranges show a higher mean consumption of SDUs per day before confinement than during confinement (*p* < 0.001), with a small effect size.

In the analysis of the differences between the different age ranges, significant differences were found for consumption frequency before confinement (F_(5.2338)_ = 33.488; *p* < 0.001; E^2^ = 0.067) and during confinement (F_(5.2338)_ = 66.884; *p* < 0.001; E^2^ = 0.125), with medium effect sizes. Significant differences were also found for the average daily alcohol consumption before confinement (F_(5.2338)_ = 10.872, *p* < 0.001; E^2^ = 0.023) and during confinement (F_(52338)_ = 5.638; *p* < 0.001; E^2^ = 0.012), with small effect sizes.

Post hoc analyses indicate that, both before and during confinement, the two lower age ranges (18–24 years and 25–29 years) showed a lower frequency of consumption than the 35 to 44 (*p* = 0.018), 45–54 (*p* < 0.001) and 55 to 64 (*p* < 0.001) age groups. Likewise, participants in the 30 to 34 and 35 to 44 age ranges showed a lower frequency of consumption than participants in the 45 to 54 (*p* < 0.001) and 55 to 64 (*p* < 0.001) age ranges. Finally, of the two higher age ranges, the older group (55–64 years) showed a higher frequency of consumption than the 45 to 54 (*p* < 0.001) and 55 to 64 (*p* < 0.001) age ranges (*p* = 0.003).

Post hoc analyses also show that, before confinement, the two lower age groups (18–24 years and 25–29 years) had a higher average daily alcohol consumption than the 35 to 44 (*p* = 0.004), 45 to 54 (*p* = 0.005), and 55 to 64 (*p* = 0.050) age groups. However, during confinement, the results were reversed, with the younger age groups (18–24 years, 25–29 years, and 30–34 years) showing a lower average daily consumption compared to the older groups, 45 to 54 years old (*p* = 0.005) and 55 to 64 years old (*p* = 0.005).

In contrast to the two previous variables, statistically significant differences were observed based on age for intensive alcohol consumption before the pandemic (F_(5.2338)_ = 19.631; *p* < 0.001; E^2^ = 0.040), but not during confinement (F_(5.2338)_ = 1.043; *p* = 0.390). In other words, during confinement, there was a generalized decrease in intensive alcohol consumption in all the age ranges, and the previous differences disappeared. Post hoc analyses show that, before the pandemic, the 18 to 29 age range had higher intensive alcohol consumption than the older age ranges (*p* < 0.05). The results also show that, as the age increased, intensive alcohol consumption decreased (*p* < 0.05).

In terms of the average number of SDUs consumed per day, significant differences were found between the different age groups before confinement (F_(5.2338)_ = 7.551; *p* < 0.001; E^2^ = 0.016) and during confinement (F_(5.2338)_ = 3.925; *p* < 0.01; E^2^ = 0.008), with small effect sizes. Post hoc analyses show that, before the pandemic, the 18 to 24 age group had higher average daily consumption of SDUs than the other groups (*p* < 0.001). However, during confinement, the 18 to 24 and 25 to 29 age groups showed a lower average daily consumption of SDUs compared to the 55- to 64-year-olds (*p* < 0.001).

**Figure 5:**
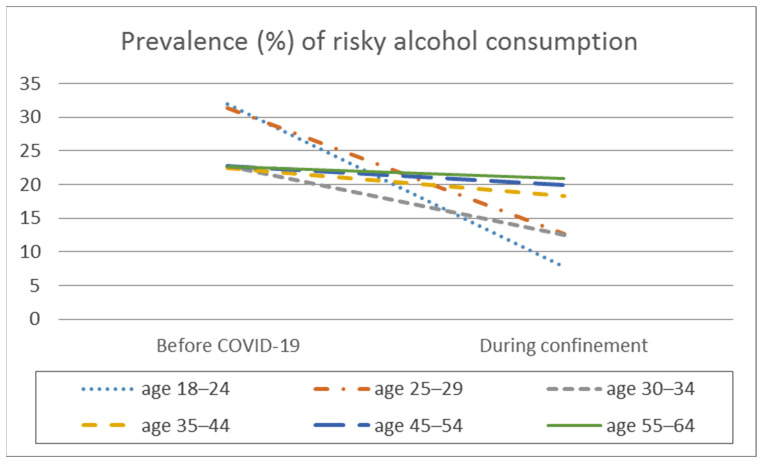
Prevalence (%) of risky alcohol consumption by age before the pandemic and during COVID-19 confinement.

Of the sample of alcohol consumers (*n* = 2345), 25.9% (*n* = 607) were classified as risky consumers before the pandemic, decreasing to 15.1% (*n* = 354) in the confinement period (Table 3, Figure 5). With the exception of the 45 to 64 age range, all the age groups showed a higher proportion of risky users before the pandemic than during confinement, with a modest decline in the 18 to 24 age group, a moderate decline in the 25 to 34 age group, and a large decline in the 35 to 44 age group (Table 3, Figure 5).

When analyzing the AUDIT-C scores in the subsample of high-risk consumers, an interaction effect was found between the sociodemographic variables (gender and living situation during confinement), the gender variable, and the four alcohol consumption variables before the pandemic and during confinement. An interaction effect of gender, age, and living situation during confinement with the frequency of alcohol consumption before the pandemic and during confinement was observed (F_(17.666)_ = 1.636; *p* < 0.05), as well as with the frequency of intensive consumption before the pandemic and during confinement (F_(17.666)_ = 2.359; *p* < 0.01). Moreover, an interaction effect was observed between average daily alcohol consumption, both before the pandemic and during confinement, and the living situation during confinement (F_(4.666)_ = 5.381; *p* < 0.001) and age (F_(5.666)_ = 6.531; *p* < 0.001). Finally, an interaction effect was observed between the mean number of SDUs per day before the pandemic and during confinement, age, and the living situation during confinement (F_(20.666)_ = 1.764; *p* < 0.05).

In the group of participants who presented risky alcohol consumption before and during confinement, their mean scores on each of the alcohol consumption variables were compared, establishing whether consumption decreased, stayed the same, or increased between the two periods (Table 4). A total of 67.4% showed a decrease in their mean AUDIT-C score, whereas 21.6% maintained the same score, and 11% increased their score. Table 4 shows the differences for the four alcohol consumption indicators.

**Figure 6:**
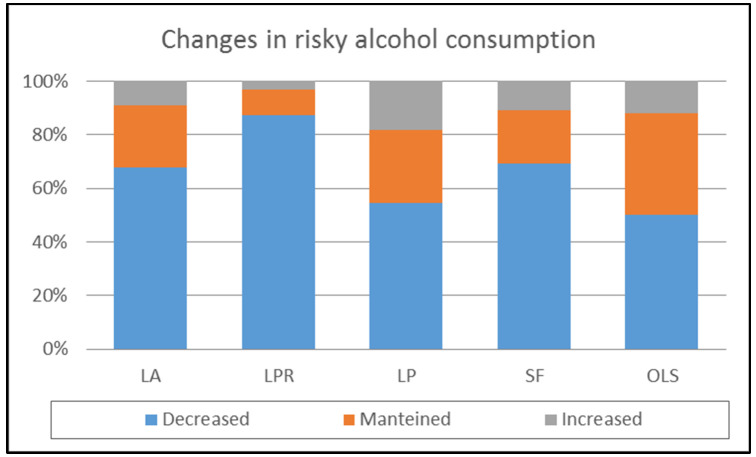
Changes in risky alcohol consumption during COVID-19 confinement considering the living situation. Note: LA = Lived alone; LPR = Lived with parents or other relatives; LP = Lived with partner; SF = Shared a flat with other people who were not the partner or family; OLS = Other living situations.

When these changes were analyzed considering the living situation of the risky consumers (Table 5, Figure 6), the largest decreases occurred in risky consumers who lived with their parents or other family members, and the largest increases occurred in those who lived alone or with a partner. Table 5 shows the differences for the alcohol consumption indicators.

In the subsample of 18- to 24-year-olds who presented risky alcohol consumption both before and during confinement, there were more females (63.5%_before_; 67.3%_during_) than males (36.5%_before_; 32.7%_during_), with an increase in the prevalence rate in females and a decline in males. Regarding the living situation, they lived alone (8.9%_before_; 14.7%_during_); lived with their parents or other relatives (51.1%_before_; 43.4%_during_); lived with a partner (10.6%_before_; 41.9%_during_); shared a flat with people who were not relatives or partners (30.5%_before_; 9.3%_during_); and had other living situations (0.6%_before_; 0%_during_).

In the subsample of 25- to 29-year-olds who presented risky alcohol consumption both before and during confinement, there were more females (59.9%_before_; 67.7%_during_) than males (40.1%_before_; 32.3%_during_), with an increase in the prevalence rate in females and a decrease in males. With regard to the living situation, they lived alone (13.3%_before_; 9.6%_during_); lived with their parents or other relatives (45.6%_before_; 24.7%_during_); lived with a partner (21.6%_before_; 43.9%_during_); and shared a flat with people who were not relatives or partners (16.3%_before_; 18.8%_during_).

In the subsample of 30- to 34-year-olds with risky alcohol consumption both before and during confinement, there were fewer females (48%_before_; 48.8%_during_) than males (52%_before_; 51.2%_during_), and the prevalence rate stayed the same in both genders. Regarding the living situation, they lived alone (23.2%_before_; 10.7%_during_); lived with their parents or other relatives (13.6%_before_; 6.7%_during_); lived with a partner (40.4%_before_; 64.6%_during_); shared a flat with people who were not relatives or partners (20.7%_before_; 12.9%_during_); and had other living situations (0.9%_before_; 1.7%_during_).

In the sub-sample of risky drinkers from 35 to 44 years old, before the pandemic there were fewer females than males, but during confinement the prevalence rate in females increased (45.4%_before_; 58.7%_during_) until reaching a higher level than in males (54.6%_before_; 41.3%_during_), who experienced a decline. Regarding the living situation, they lived alone (17.6%_before_; 14%_during_); lived with their parents or other relatives (10.8%_before_; 8.6%_during_); lived with a partner (64.1%_before_; 65.9%_during_); shared a flat with people who were not relatives or partners (5%_before_; 4.4%_during_); and had other living situations (2.6%_before_; 4.9%_during_).

In the subsample of risky drinkers from 45 to 54 years old, before the pandemic the prevalence rate in females and males was similar, but during confinement it increased in females (50.7%_before_; 64.4%_during_) until reaching higher levels than in males (49.3%_before_; 35.6%_during_), who experienced a decline. Regarding their living situation, they lived alone (18.5%_before_; 6.3%_during_); lived with their parents or other relatives (12.2%_before_; 20.9%_during_); lived with a partner (62.3%_before_; 66.9%_during_); shared a flat with people who were not relatives or partners (1.6%_before_; 0.9%_during_); and had other living situations (1.8%_before_; 2.7%_during_).

In the subsample of risky drinkers from 55 to 64 years old, both before and during confinement, there was a higher prevalence of females (35.9%_before_; 45.2%_during_) than males (64.1%_before_; 54.8%_during_); however, there was an increase in the prevalence in females and a decrease in males. Regarding the living situation, they lived alone (15.4%_before_; 9.3%_during_); lived with their parents or other relatives (15.4%_before_; 30.2%_during_); lived with a partner (67.3%_before_; 66.2%_during_); shared a flat with people who were not relatives or partners (4.8%_before_; 3.7%_during_); and had other living situations (4.8%_before_; 8.3%_during_).

## 4. Discussion

The aim of this study was to characterize changes in the prevalence and pattern of alcohol consumption during the COVID-19 confinement in the Spanish adult population. These changes were analyzed according to age and, in the subsample of risky consumers, according to the living situation. In general terms, the findings indicate considerable heterogeneity in consumption practices among the age groups and the existence of interactions with gender and the living situation during confinement. Likewise, the living situation during confinement acts as a protective or risk factor in at-risk consumers, favoring decreases or increases. Other similar studies contemplate less broad age ranges and have smaller sample sizes that make a segmented analysis difficult, thus limiting the ability to show different realities depending on individuals’ life cycle stage and circumstances. Therefore, the findings of the present study make a noteworthy contribution to the literature on alcohol consumption, both in periods of crisis and in periods of normality, and they are a reference for future research that monitors post-COVID alcohol consumption.

The first conclusion that can be drawn from our findings is that, during confinement, the percentage of people who consumed alcohol decreased, as did the frequency and indicators of consumption and risky drinking. Approximately five out of ten people who consumed alcohol before the pandemic reduced their consumption during confinement, whereas a disturbing 16.2% increased their consumption. Other studies conducted during confinement indicate that about 14% of alcohol drinkers increased their consumption during this period [42,43,44]

The second conclusion is that being a young adult was a strong predictor of decreased alcohol consumption during confinement. These findings are consistent with other studies [10,11,15,16,17,18] that report a greater decline in the prevalence and frequency of drinking in people from 18 to 29 years old. This greater decrease in all the indicators of alcohol consumption in the youngest adults, compared to older adults, can be explained, at least partially, by limitations on social drinking opportunities due to the closure of venues for young people (discotheques, festivals, and pubs) where they were used to drinking alcohol regularly [44], in contrast to older drinkers, whose consumption is more associated with the home [28,44,45]. In short, the environmental contingencies resulting from the COVID-19 restrictions favored the general reduction in alcohol consumption in young people [43,44]; therefore, the reduction is due more to circumstance than to a reasoned decision. It remains to be seen whether this consumption changed after lifting the restrictions and sanctions [44]. In addition, the limited changes in the drinking pattern observed in older adults (45–64 years of age) may be related to various strategies for rationalizing consumption [46]. Confinement has indirectly shown the benefits of strategies to regulate consumption spaces and the restriction of alcohol consumption itself, especially in young people and in contexts associated with leisure.

The third conclusion is that, during confinement, despite the generalized decrease in alcohol consumption, there was a higher percentage of people who increased their mean AUDIT-C score and the frequency of their consumption. Specifically, the percentage of those who consumed alcohol four or more days a week was 1.5 times greater during confinement (11.54% vs. 17.57%) and more pronounced in the 18 to 44 age group. This finding nuances the decreases reported in young adults, indicating that a portion of consumers showed an increase in the frequency of alcohol consumption [21,22,23]. These cases could be related to the use of alcohol as a coping mechanism to deal with the stress associated with social isolation, insecurity, and economic hardship resulting from the COVID-19 pandemic [1,19,30,44,47,48]. Because this situation lasted for more than 12 months, there is a risk that alcohol consumption as a strategy for coping with the crisis and associated stressful events may have favored the development of alcohol-related problems, given that several studies have established this association [19]. This situation may have increased the vulnerability of drinkers with a higher level of dependence [10,29], increasing their consumption frequency.

Focusing on the subsample of risky drinkers, during confinement this group decreased from 25.9% to 15.1%. This decrease can be seen in the percentage of risky consumers before the pandemic who decreased their mean score on the AUDIT-C (67.6%) and on the other indicators, especially the frequency of intensive use (65.7%). However, 11% increased their mean AUDIT-C scores, and 15.1% increased the frequency of consumption. In terms of age, the percentage of risky drinkers before the pandemic was around 23% in the 30 to 64 age range, whereas in the 18 to 29 age range, it was around 32%. In contrast to other studies that indicated that risky consumption especially decreased in people from 18 to 29 years old during confinement [10,11,15,16,17,18], in our study, the greatest decreases were observed in the 35 to 44 age group, followed moderately by the 25 to 34 age group and, finally, by the 18 to 24 age group, but only slightly.

Regarding the other study variables, gender and living situation before and during confinement, age, and living situation had an effect on the frequency of alcohol consumption and heavy drinking. Likewise, there is an interaction effect between the average daily alcohol consumption, both before the pandemic and during confinement, and the living situation during confinement and age, as well as between the average number of SDUs per day before the pandemic and during confinement and age and the living situation. This finding highlights the underlying complexity of the alcohol consumption patterns before and during the pandemic as well as the changes observed in the latter period.

Some studies conducted in the COVID-19 period have pointed out the relevance of gender [22,27], including a previous and complementary study to this one. In line with the findings, there are studies that show a reduction in the alcohol consumption gap between males and females [49,50,51,52], particularly in younger females [52,53]. In this regard, in our study, we found that this phenomenon was confirmed and that young females between 18 and 29 years old had a higher rate of risky alcohol consumption than males, both before and during confinement. In intermediate adult ages (30–54 years), the percentages were similar before the pandemic, but during confinement, there was a decrease in the percentage of males, whereas the percentage of females increased. Only in the 54 to 64 age range, females had a lower prevalence than males both before and during confinement, although it increased during confinement, whereas it decreased in males. According to the study by Canfield et al. [30], this latter group of females over 50 years old has less risky alcohol consumption than younger age groups.

Finally, in the subsample of risky consumers, the role of the living situation was analyzed, showing that it was relevant when interacting with age, but also on its own. On the one hand, the largest decreases in the mean AUDIT-C score and on the four indicators of alcohol consumption were observed in at-risk consumers living with their parents or other relatives. On the other hand, the greatest increases were found in at-risk consumers who lived alone or with a partner and those who indicated another living situation. There was also a high percentage of at-risk consumers who maintained their consumption pattern, regardless of their family situation.

Analyzing the living situation in the different age ranges in the subsample of risky consumers, we can highlight that there was an increase in the percentage of risky consumers during confinement among those who lived with a partner in the younger age ranges: in the 18 to 24 age group, it quadrupled; in the 25 to 29 age group, it doubled; and in the 30 to 34 age group, it was multiplied by a factor of 1.6. There are studies that indicate that drinking alcohol with a partner at home is a practice related to risky consumption, but that it is more predominant in older age groups [30]. An increase was also observed in those who lived alone in the 18 to 25 age range, with the percentage multiplied by a factor of 1.7, although this may be related to isolation or loneliness [34]. These phenomena need to be analyzed in further studies.

For the situation of living with parents or other relatives, an increase was observed in the higher age ranges, given that it was 1.7 times higher in the 45 to 54 age range and twice as high in the 55 to 64 age range. An increase in stress stemming from constant care at home during confinement could be a plausible explanation for these increases if alcohol consumption was used as a strategy for coping with stress [31,33].

Finally, it should be noted that the prevalence rates for risky consumption in the home in participants living with a partner were very high in the older age ranges (35–64 years), between 60–70%, both before and during confinement. In the younger age ranges, an increase was observed during confinement, approaching the percentages in the immediately younger age ranges. This suggests a process associated with changes in drinking contexts from outside to inside the home as age increases.

## 5. Conclusions

In summary, the findings indicate that alcohol consumption during confinement decreased in the adult population in all the age ranges, being more pronounced in adults from 18 to 29 years old and less noticeable with increasing age. However, around 16% of consumers increased their consumption during confinement, indicating heterogeneous patterns in the changes produced. In addition to age, gender and living situation are important explanatory variables. Among high-risk users, the percentage of females was higher than that of males at younger ages, similar at intermediate ages, and lower at older ages. However, in almost all the age ranges, there was an increase in the percentage of females with risky consumption, whereas it decreased in males. With regard to the living situation, the greatest decreases in alcohol consumption occurred in those who lived with their parents or other relatives, and the greatest increases in those who lived alone or with a partner. In sum, the life cycle stage and living situation are determining factors in explaining alcohol consumption patterns and the changes that occur.

Given this heterogeneity in alcohol consumption practices based on the variables analyzed, several recommendations can be made from a prevention perspective:

(a) Strategies to regulate alcohol consumption and restrict access to alcohol in contexts associated with young people’s leisure activities would be effective [11], especially in adults from 18 to 34 years old. In addition, considering that the rate of risky consumers in the youngest age range is around 32%, which is nine percentage points higher than what was found for other age groups, prevention, early detection, and treatment interventions should be promoted in this population group, especially for females.
(b) In the older alcohol-drinking population, the consumption pattern did not vary substantially, which, in line with the existing literature, suggests that the main context for drinking is the home. Therefore, it would be advisable to carry out awareness campaigns about the effects and risks of alcohol consumption at home, focusing especially on the target population aged 45 to 64.
(c) Among at-risk consumers from 35 to 64 years old, 60–70% live with a partner. Cohabitation and living alone are the situations that have shown the greatest increases in alcohol consumption during confinement. Therefore, awareness campaigns should be carried out related to alcohol consumption in couples within the home, intensifying in situations of crisis or periods similar to the COVID-19 pandemic.
(d) Living in the family setting is a protective factor for high-risk consumers, given that there was a significant decrease in all the consumption indicators. This fact demonstrates the importance of the role of the family as a direct or indirect agent for prevention and in treatment processes.

Some of the limitations of this study are the possible errors in coverage, the randomness of the sample, and the response rate, due to the use of an online survey. In any case, actions to compensate for these errors were carried out (see Design and Population sections above). Although our sample was large, it cannot be considered representative of the Spanish population. Therefore, the findings should be generalized with caution. Future studies should consider the role of income level, the presence of psychopathological symptomatology, and coping styles. These variables seem to be relevant in crisis situations and could offer a better understanding of change processes in alcohol consumption, especially in at-risk drinkers. A limitation to consider in this study is the use of online self-reports, although self-reports are considered valid and reliable strategies because they guarantee the anonymity of the participant and the confidentiality of the data [54]. Even so, it should be noted that self-reports of changes in alcohol consumption are subjective perceptions and may be influenced by social desirability biases [55], by the social and cultural norms of each autonomous community in Spain, and by the pressure and influence of the media on these perceptions [56]. Although participants reported on their consumption before the pandemic, the lack of a pre-COVID-19 measure is important, limiting causal interpretations. Longitudinal studies would allow us to find out whether this change lasts over time.

## Figures and Tables

**Table 1:** Changes in alcohol consumption during confinement (*n* = 2282).

		Age						
		Total % (*n*)	18–24 % (*n*)	25–29 % (*n*)	30–34 % (*n*)	35–44 % (*n*)	45–54 % (*n*)	55–64 % (*n*)
AUDIT-C	Decreased	49.5 (1101)	73 (301)	62.6 (309)	47.6 (154)	34.7 (172)	31.8 (120)	24.7 (45)
	Maintained	33.8 (810)	17.7 (73)	26.8 (132)	36.8 (119)	42.1 (208)	46.8 (177)	55.5 (101)
	Increased	16.7 (371)	9.2 (38)	10.5 (52)	15.4 (50)	23 (114)	21.5 (81)	19.6 (36)
Frequency of consumption	Decreased	36.2 (827)	59.5 (245)	45 (222)	34.1 (110)	24.1 (119)	24.9 (94)	20.3 (37)
	Maintained	45.4 (1037)	29.4 (121)	41 (202)	45.5 (147)	50.6 (250)	54.8 (207)	60.4 (110)
	Increased	18.3 (418)	11.1 (46)	14 (69)	20.4 (66)	25.3 (125)	20.4 (77)	19.2 (35)
Average daily consumption	Decreased	14.2 (323)	26.9 (111)	21 (104)	11.4 (37)	8.5 (42)	5.8 (22)	3.8 (7)
	Maintained	82.8 (1890)	71.6 (295)	75.7 (373)	85.8 (277)	88.7 (438)	89.4 (338)	92.9 (169)
	Increased	3 (69)	1.4 (6)	3.2 (16)	2.8 (9)	2.8 (14)	4.7 (18)	3.2 (6)
Frequency of intensive consumption	Decreased	29.3 (668)	46.6 (192)	39.8 (196)	29.5 (95)	22.2 (110)	16.2 (61)	7.6 (14)
	Maintained	66.4 (1515)	49.5 (204)	57.6 (284)	65 (210)	72.9 (360)	78.8 (298)	87.4 (159)
	Increased	4.3 (99)	3.9 (16)	2.6 (13)	5.6 (18)	4.8 (24)	5.1 (19)	4.9 (9)
Average SDUs ^1^ per day	Decreased	7.5 (171)	17.5 (72)	8.5 (42)	4.6 (15)	3.8 (19)	4.5 (17)	3.3 (6)
	Maintained	88.6 (2021)	80.3 (331)	88.6 (437)	92 (297)	91.1 (450)	89.4 (338)	92.3 (168)
	Increased	3.9 (90)	2.2 (9)	2.8 (14)	3.4 (11)	5.1 (25)	6.1 (23)	4.3 (8)

^1^ Standard Drink Units.

**Table 2:** Alcohol consumption patterns before and during confinement as a function of age (*n* = 2282).

		Before Confinement M (SD ^1^)	During Confinement M (SD ^1^)	*t*	*p*	*d*
Frequency of consumption	18–24	2.01 (0.846)	1.22 (1.245)	13.864	0.001	−0.722
	25–29	2.05 (0.860)	1.61 (1.228)	8.685	0.001	−0.378
	30–34	2.17 (0.871)	1.94 (1.280)	4.484	0.001	−0.198
	35–44	2.25 (0.997)	2.19 (1.313)	1.375	0.170	
	45–54	2.53 (1.009)	2.46 (1.266)	1.480	0.140	
	55–64	2.83 (1.049)	2.75 (1.273)	0.241	0.241	
Average daily consumption	18–24	0.42 (0.733)	0.06 (0.278)	10.043	0.001	−0.619
	25–29	0.32 (0.630)	0.09 (0.331)	8.140	0.001	−0.440
	30–34	0.27 (0.635)	0.13 (0.372)	4.465	0.001	−0.268
	35–44	0.19 (0.504)	0.12 (0.440)	3.375	0.001	−0.139
	45–54	0.18 (0.454)	0.16 (0.437)	0.810	0.418	
	55–64	0.19 (0.427)	0.21 (0.571)	−0.873	0.384	
Frequency of intensive consumption	18–24	0.82 (0.889)	0.20 (0.603)	13.574	0.001	−0.591
	25–29	0.86 (0.965)	0.26 (0.645)	14.170	0.001	−0.588
	30–34	0.63 (0.846)	0.26 (0.664)	8.512	0.001	−0.416
	35–44	0.55 (0.859)	0.30 (0.815)	7.846	0.001	−0.332
	45–54	0.46 (0.813)	0.30 (0.725)	4.206	0.001	−0.207
	55–64	0.33 (0.778)	0.26 (0.774)	1.658	0.099	
Average SDUs ^2^ per day	18–24	0.30 (0.688)	0.08 (0.405)	5.953	0.001	−0.246
	25–29	0.16 (0.512)	0.08 (0.381)	3.527	0.001	−0.148
	30–34	0.14 (0.498)	0.12 (0.467)	1.056	0.292	
	35–44	0.11 (0.407)	0.13 (0.482)	−1.368	0.172	
	45–54	0.15 (0.413)	0.16 (0.454)	−0.683	0.495	
	55–64	0.17 (0.454)	0.21 (0.523)	−1.630	0.105	

^1^ Standard Deviation; ^2^ Standard Drink Units.

**Table 3:** Proportion of risky alcohol consumption by age before and during confinement (*n* = 2345).

Age	*n*	Before Confinement % (*n*)	During Confinement % (*n*)	X^2^_MN_^1^	*p*	*Phi*
18–24	400	32 (128)	7.8 (31)	86.523	0.001	0.282
25–29	454	31.3 (142)	12.6 (57)	62.151	0.001	0.318
30–34	338	22.8 (77)	12.5 (43)	21.121	0.001	0.449
35–44	531	22.5 (119)	18.3 (97)	6.782	0.017	0.556
45–54	402	22.7 (91)	19.9 (80)	2.485	0.182	
55–64	220	22.7 (50)	20.9 (46)	0.666	0.541	

^1^ McNemar’s test.

**Table 4:** Changes in alcohol consumption during confinement in risk consumers (*n* = 607).

Alcohol Variables	Decreased % (n)	Maintained % (n)	Increased % (n)
AUDIT-C	67.4 (409)	21.6 (131)	11 (67)
Frequency of consumption	39 (237)	46 (279)	15.1 (91)
Average daily consumption	34.7 (210)	59 (358)	6.4 (39)
Frequency of intensive consumption	65.7 (398)	28.5 (173)	5.8 (35)
Average SDUs ^1^ per day	14 (84)	76.5 (465)	9.5 (58)

^1^ Standard Drink Units.

**Table 5:** Changes in alcohol consumption according to the living situation during confinement in risky consumers (*n* = 607).

		Living Situation				
		LA % (*n*)	LPR % (*n*)	LP % (*n*)	FS % (*n*)	OLS % (*n*)
AUDIT-C	Decreased	66.6 (48)	87.4 (176)	54.4 (131)	63.9 (23)	50 (28)
	Maintained	23,6 (17)	9.7 (20)	27.4 (66)	19.4 (7)	39.3 (22)
	Increased	9.7 (7)	3 (6)	18.2 (44)	16.6 (6)	10.7 (6)
Frequency of consumption	Decreased	45.8 (33)	59.9 (121)	21.2 (51)	44.1 (16)	26.8 (15)
	Maintained	43.1 (31)	32.2 (65)	53.9 (130)	50 (18)	62.5 (35)
	Increased	11.1 (8)	7.9 (16)	24.9 (60)	6 (2)	10.1 (6)
Average daily consumption	Decreased	41.6 (30)	51 (103)	21.2 (51)	22.2 (8)	33.3 (17)
	Maintained	55.6 (40)	46 (93)	69.7 (168)	69.4 (25)	54.1 (33)
	Increased	2.7 (2)	3 (6)	9.1 (22)	8.3 (3)	10.7 (6)
Frequency of intensive consumption	Decreased	56.9 (41)	86.6 (175)	54.4 (131)	63.4 (23)	50 (28)
	Maintained	33.3 (24)	12 (24)	36.9 (89)	30 (11)	46.4 (26)
	Increased	9.7 (7)	1.5 (3)	8.7 (21)	6.7 (2)	3.6 (2)
Average SDUs per day	Decreased	10.9 (7)	22.7 (46)	8.3 (20)	16.7 (6)	8.9 (5)
	Maintained	83.9 (61)	73.8 (149)	76.8 (185)	76.7 (27)	73.2 (41)
	Increased	5.3 (4)	3.5 (7)	14.9 (36)	6.7 (3)	17.9 (10)

Note: LA = lived alone; LPR = lived with parents or other relatives; LP = lived with partner; SF = shared a flat with other people who were not the partner or a relative; OLS = other living situations. SDUs = Standard Drink Units.
